# Benchtop and bedside validation of a low-cost programmable cortical stimulator in a testbed for bi-directional brain-computer-interface research

**DOI:** 10.3389/fnins.2022.1075971

**Published:** 2023-01-12

**Authors:** Won Joon Sohn, Jeffrey Lim, Po T. Wang, Haoran Pu, Omid Malekzadeh-Arasteh, Susan J. Shaw, Michelle Armacost, Hui Gong, Spencer Kellis, Richard A. Andersen, Charles Y. Liu, Payam Heydari, Zoran Nenadic, An H. Do

**Affiliations:** ^1^Department of Neurology, University of California, Irvine, Irvine, CA, United States; ^2^Department of Biomedical Engineering, University of California, Irvine, Irvine, CA, United States; ^3^Department of Electrical Engineering and Computer Science, University of California, Irvine, Irvine, CA, United States; ^4^Department of Neurology, Rancho Los Amigos National Rehabilitation Center, Downey, CA, United States; ^5^Department of Neurology, University of Southern California, Los Angeles, CA, United States; ^6^Division of Biology and Biological Engineering, California Institute of Technology, Pasadena, CA, United States; ^7^Department of Neurosurgery, Rancho Los Amigos National Rehabilitation Center, Downey, CA, United States; ^8^Department of Neurological Surgery, University of Southern California, Los Angeles, CA, United States

**Keywords:** brain stimulator, brain computer interface, bi-directional BCI (BD-BCI), miniaturized stimulator, neuroprosthetics, cortical stimulator

## Abstract

**Introduction:**

Bi-directional brain-computer interfaces (BD-BCI) to restore movement and sensation must achieve concurrent operation of recording and decoding of motor commands from the brain and stimulating the brain with somatosensory feedback.

**Methods:**

A custom programmable direct cortical stimulator (DCS) capable of eliciting artificial sensorimotor response was integrated into an embedded BCI system to form a safe, independent, wireless, and battery powered testbed to explore BD-BCI concepts at a low cost. The BD-BCI stimulator output was tested in phantom brain tissue by assessing its ability to deliver electrical stimulation equivalent to an FDA-approved commercial electrical cortical stimulator. Subsequently, the stimulator was tested in an epilepsy patient with subcortical electrocorticographic (ECoG) implants covering the sensorimotor cortex to assess its ability to elicit equivalent responses as the FDA-approved counterpart. Additional safety features (impedance monitoring, artifact mitigation, and passive and active charge balancing mechanisms) were also implemeneted and tested in phantom brain tissue. Finally, concurrent operation with interleaved stimulation and BCI decoding was tested in a phantom brain as a proof-of-concept operation of BD-BCI system.

**Results:**

The benchtop prototype BD-BCI stimulator's basic output features (current amplitude, pulse frequency, pulse width, train duration) were validated by demonstrating the output-equivalency to an FDA-approved commercial cortical electrical stimulator (*R*^2^ > 0.99). Charge-neutral stimulation was demonstrated with pulse-width modulation-based correction algorithm preventing steady state voltage deviation. Artifact mitigation achieved a 64.5% peak voltage reduction. Highly accurate impedance monitoring was achieved with *R*^2^ > 0.99 between measured and actual impedance, which in-turn enabled accurate charge density monitoring. An online BCI decoding accuracy of 93.2% between instructional cues and decoded states was achieved while delivering interleaved stimulation. The brain stimulation mapping *via* ECoG grids in an epilepsy patient showed that the two stimulators elicit equivalent responses.

**Significance:**

This study demonstrates clinical validation of a fully-programmable electrical stimulator, integrated into an embedded BCI system. This low-cost BD-BCI system is safe and readily applicable as a testbed for BD-BCI research. In particular, it provides an all-inclusive hardware platform that approximates the limitations in a near-future implantable BD-BCI. This successful benchtop/human validation of the programmable electrical stimulator in a BD-BCI system is a critical milestone toward fully-implantable BD-BCI systems.

## Introduction

Brain computer interfaces (BCIs) allows users to directly translate their motor intention measured from electrophysiological or other signals of the brain to control external devices to carry out desired actions. The advancement in electrophysiological signal acquisition and decoding have demonstrated promising results in motor control of robotic limbs or muscle stimulation through one-way communication between brain and external devices (Wodlinger et al., 2014; Bouton et al., 2016). In BCI applications where visual feedback is solely sufficient, such as keyboard typing, open-loop, uni-directional BCI may be sufficient. However, real-life movement invariably involves continuous interaction with external objects and the environment. In human motor control, the role of sensory feedback in movement planning, control, and motor learning is known to play an integral part necessitating complex sensorimotor integration (Wolpert et al., 1995). The theory of optimal feedback control (Wolpert, 1997) affirms that humans rely on cost and rewards (Todorov and Jordan, 2002; O'Sullivan et al., 2009), internal models (Shadmehr and Mussa-Ivaldi, 1994; Kawato, 1999), optimal feedback-driven policy (Körding, 2007), and state estimation (Ernst and Banks, 2002; Körding, 2007), all of which demand somatosensory feedback as a crucial component of normal motor control. Physiological studies corroborate that the loss of somatosensation causes severe deficits in motor control (Rothwell et al., 1982; Sainburg et al., 1993; Gordon et al., 1995). Therefore, an important challenge for BCI development has been to realize a bi-directional BCI (BD-BCI) technologies that deliver sensory information simultaneously with motor decoding.

BD-BCI research initially largely focused on characterizing how various stimulation parameters could evoke different modalities of sensation (Johnson et al., 2013; Cronin et al., 2016; Collins et al., 2017; Hiremath et al., 2017; Lee et al., 2018; Caldwell et al., 2019). More recently, a closed-loop BD-BCI demonstrated improved prosthetic arm motor control (Raspopovic et al., 2014; Weiss et al., 2018; Flesher et al., 2021). However, operations of the existing BD-BCI systems are limited to a laboratory setting where the systems run on bulky non-mobile work station computers, data acquisition systems, and commercial stimulators. The reliance on such bulky systems is due to most invasive BCIs requiring high performance computing to undertake the signal processing on extremely high dimensional neuronal population data. In order for BD-BCIs to become practical, all of the above components must be integrated into a special purpose and compact form factor with full programmability. Most importantly, it must be shown to be safe—specifically equivalent to predicate FDA-approved cortical stimulators. As a critical step toward this goal, we propose a fully integrated and compact BD-BCI benchtop prototype with rigorous comparison of its sensory stimulation module against an FDA-approved cortical stimulator.

### A vision for a fully-implantable bi-directional BCI

**Figure 1:**
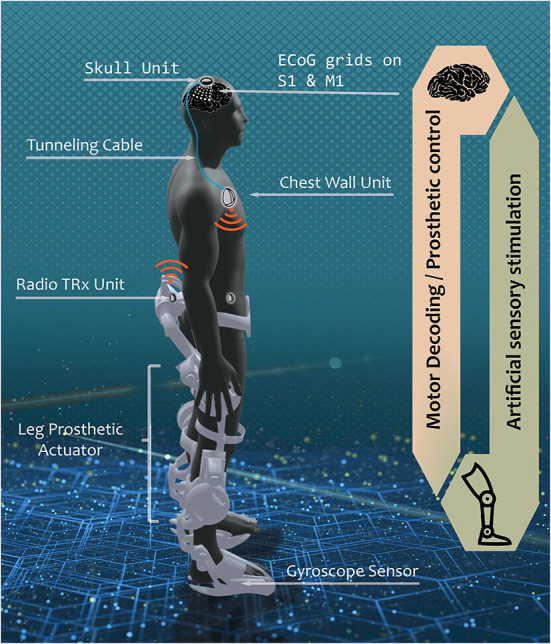
Envisioned fully implantable BD-BCI system. In a hypothetical scenario where the BD-BCI system is implanted in persons with severe spinal cord injury, a downstream motor signal from ECoG electrodes on the primary motor cortex (M1) is amplified, multiplexed, and digitized by the skull unit (SU) to bypass the damaged spinal cord connection through tunneling cable to reach the chest wall unit (CWU). The signal is decoded in the CWU which wirelessly actuates leg prosthetic for walking. Upstream sensation of walking is encoded with gyroscope sensor. The sensory information is wirelessly transmitted to the CWU. The CWU converts the received kinematic info into electrical pulse train which travels *via* the tunneling cable, multiplexed in the SU to stimulate the desired loci in the primary sensory cortex (S1) to elicit artificial sensation. The current study focuses on the development of upstream electrical stimulator, encompassing the CWU analog to the SU analog.

Our envisioned grand scheme of a fully-implantable BD-BCI system is a hypothetical scenario where a person with spinal cord injury (SCI) is implanted with the skull unit (SU) and the chest wall unit (CWU) connected by a tunneling cable subcutaneously (Figure 1). The ECoG electrodes are implanted over sensorimotor cortex and the downstream motor signal from the motor cortex is amplified, multiplexed, and digitized in the SU which is then decoded in the CWU. The decoded motor commands are wirelessly transmitted to leg prosthesis to actuate walking. Sensors within the prosthesis encode leg kinematics and transmit wirelessly back to the CWU, where the encoded sensory information will be converted into electrical stimulation patterns. The electrical stimulation will be delivered to the sensory brain *via* the tunneling cable, multiplexed in the SU to target specific loci, thereby eliciting artificial leg sensation. It should be emphasized that a similar setup can be applied toward other applications, such as upper extremity movements and sensation. It should also be noted that the more power-hungry processes, including signal analysis and wireless transmission, are performed in the CWU to minimize exposure of the brain to heat and wireless signals.

Toward this vision, we previously developed a benchtop BCI system for decoding motor commands from ECoG signals (Wang et al., 2019), but without the above sensory feedback component. In this study, the electrical cortical stimulator was designed and integrated into the existing unidirectional BCI system (Wang et al., 2019) and validated to be equivalent to commercial FDA approved stimulators at benchtop and bedside. Furthermore, the successful integration of a fully programmable stimulator and decoder into a single embedded system was demonstrated in the benchtop online BD-BCI operation. This BD-BCI development milestone provides a testbed platform which safely enables validation of various BD-BCI concepts in human with realistic, approximate constraints of a future fully implantable system. This “board level” prototype thus provides an analog of a future implantable BD-BCI which approximates the expected hardware resources and computational limits.

## Methods

### System overview

**Figure 2:**
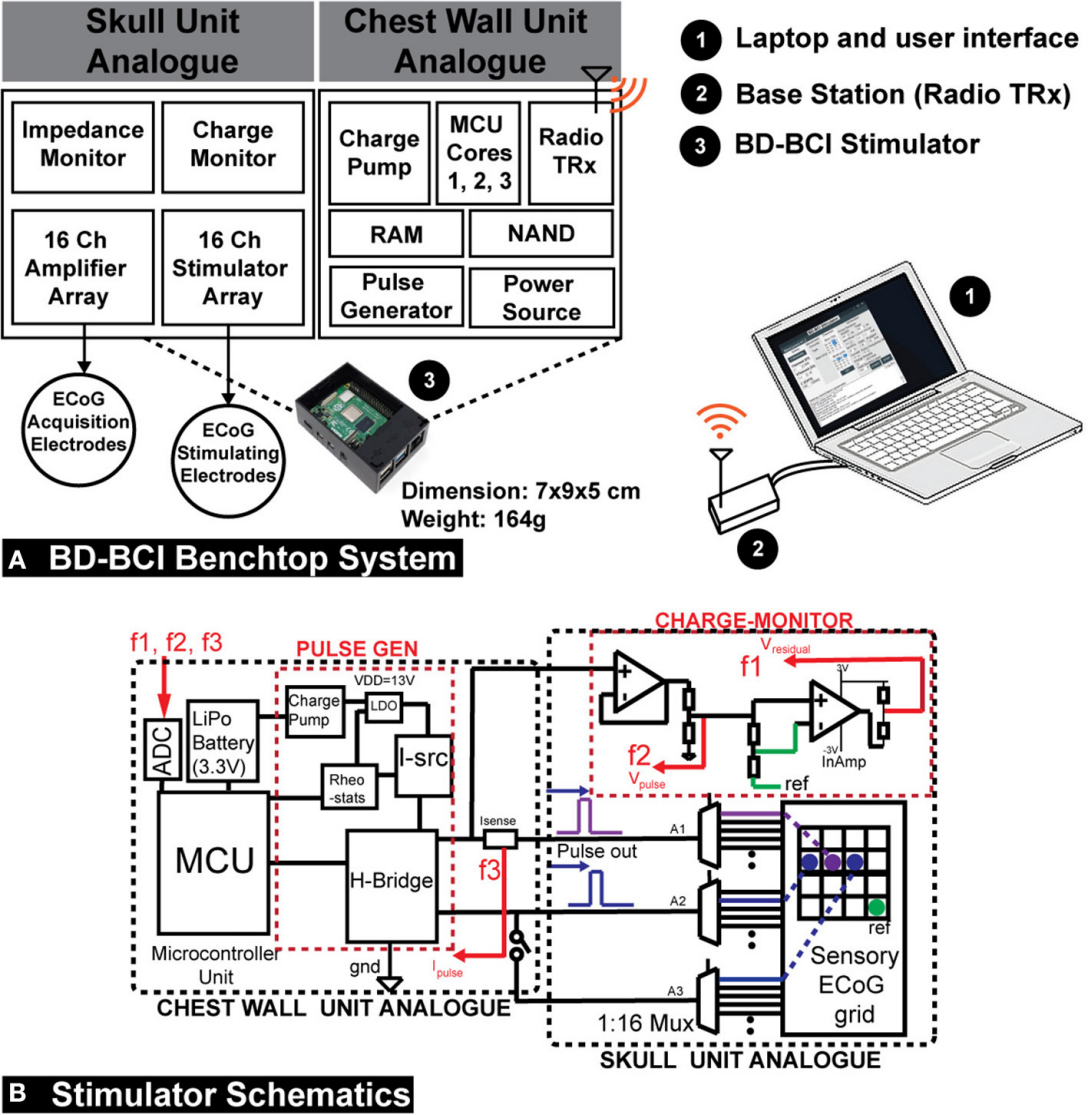
**(A)** A diagram of the operation of the prototype implantable BD-BCI stimulator. Compressing the structure of the envisioned fully implantable BCI system on a custom printed circuit board, the CWU analog supplies electrical pulse trains to the SU analog which has a connector interfaced with ECoG electrodes. The CWU, composed of 3 microcontroller (MCU) cores and supporting components, performs all necessary processing to control electrical stimulation. Cores 1 and 2 are used for BCI functions while Core 3 is dedicated to controlling the stimulator module. The base station is used to wirelessly configure the implantable BD-BCI stimulator through medical (ISM) radio band. The BD-BCI stimulator is powered by a rechargeable battery which can be charged wirelessly. The prototype fully-implantable system has a weight of 164 g, and the case's dimension is 7 x 9 x 5 cm which is equivalent to the size of a Raspberry Pi. **(B)** Left: The design schematic of the stimulator. The CWU comprises microcontroller, H-bridge, current source(I-src), charge pump, LDO, digital rheostats, current sensor, and battery. SU analog comprises the three multiplexers (A1–A3) and charge-monitor. ECoG electrodes are plugged into standard touchproof jacks. F1, F2, and F3: feedback signals for voltage and impedance monitoring. Isense, current sensor; InAmp, Instrumentation amplifier; PULSE GEN, pulse generator; REF, reference electrode.

The electrical stimulator (BD-BCI stimulator) is integrated into a miniaturized benchtop fully-implantable BCI system. The CWU and SU analog are modularized and custom-designed on the printed circuit board (PCB) (Figure 2A). The CWU analog supplies electrical pulses to the SU analog which is interfaced with ECoG electrode grids. The CWU is composed of multiple microcontroller cores (three 48 MHz ARM Cortex-M0+ microcontrollers; Microchip, Chandler, AZ; 2 of which are used for BCI decoding operation, and 1 which is dedicated to controlling the stimulator hardware) and supporting components to maximize the resource and interfacing with peripherals such as 32-channel commercial bioamplifer integrated circuit with integrated multiplexer and 16-bit analog-to-digital converter (ADC) (Intan Technology, Santa Monica, CA), MedRadio band radio transceiver (TRX) (HOPE Microelectronics, Xili, Shenzhen, China), memory (two 512-KiB FRAM; Cypress Semiconductor, San Jose, CA), and storage modules (512-MiB NAND flash memory; Micron, Boise, ID). The wireless control and data transmission between the base station and the TRX in the CWU (Figure 2A) are designed to comply with Federal Communication Commission designated Medical Device Radiocommunications Service (FCC MedRadio; 47 C.F.R., 2017) for implantable medical devices. The benchtop BD-BCI system is battery-powered and can be charged wirelessly (Qi 2.1 standard). Additional BCI system details are in Wang et al. (2019).

### Stimulator hardware and software design

The stimulator was designed to be controlled by one of the two microcontroller (MCU) cores of the previously developed benchtop system for a fully-implantable BCI interface (Wang et al., 2019). Biphasic square pulses were generated by a digitally controlled H-bridge (Texas Instrument, Dallas, TX). The H-bridge was driven by a current source (Linear Technology, Norwood, MA), two cascaded charge pumps (Maxim Integrated, San Jose, CA), and an LDO (Texas Instrument, Dallas, TX), which collectively boost voltage from 3.3V (Vcc) to 13V to meet the current demand (Figure 2B). The current level was digitally controlled by adjusting input parameters for the current source. A pair of 1:16 multiplexers (A1-A2) (Analog Devices, Norwood, MA) enabled a selection of the electrode pair for a bipolar stimulation from a pool of up to 16 ECoG electrodes. A third 1:16 multiplexer (A3) enabled an extra electrode to be optionally selected to form a triple-pole artifact mitigation path (see Section: “Artifact mitigation”). The microcontroller Timer/Counter for Control Applications (TCC) peripheral was used to trigger stimulation with precise digital control of the pulse duration, frequency of stimulation, and train duration. A breakout-board-interface with industrial standard 1.5 mm touchproof connectors was designed to facilitate connection from the BD-BCI prototype stimulator and amplifier array to ECoG electrodes, and accommodates up to 16 stimulation electrodes and 16 recording channels, reference and the ground (GND) connection. A kill-switch allowed for emergency power shut off.

The above circuit design was implemented as a PCB using CAD software. Specifically, the stimulator circuitry layout was split across 4 modular PCBs to facilitate debugging: (1) Mainboard which includes the MCUs, memory, storage, and transceivers. (2) Stimulation and charge balancing board which includes the circuit related to pulse generation. (3) Multiplexer board. (4) Touch-proof connector break-out board. The high-density flat flexible cables (FFC) are used to connect between the boards.

Similar to our prior BCI prototype, all stimulation control and data transmission for the BD-BCI system were wireless. Specifically, the base station software was designed with graphical user interface (GUI) written in Visual C# that enabled full control of stimulation parameters including pulse widths, pulse frequency, train duration, current level, and channel selection. All of the BCI decoding functions described in Wang et al. (2019) remained in place and was integrated with the stimulation capability (described in further detail below). All commands were transmitted wirelessly *via* the TRX.

### Design and validation of basic stimulator functions

It is well known that somatosensory percepts and motor responses (e.g., muscle contraction) can be elicited with electrical stimulation of the primary sensory (S1) and motor cortices (M1), respectively (Penfield and Boldrey, 1937; Libet, 1993). Furthermore, adjusting stimulation parameters such as current amplitude, pulse frequency, and pulse width can change the type or quality of sensation (Johnson et al., 2013; Cronin et al., 2016; Hiremath et al., 2017; Caldwell et al., 2019) and provide a sense of ownership of an artificial limb (Collins et al., 2017). Therefore, our BD-BCI stimulator was designed to have full control over pulse frequency, pulse duration, train duration, and current level so as to potentially deliver a wide variety of evoked sensory percepts. The output specifications of the commercial stimulator are summarized in **Table 2A**. The BD-BCI stimulator was designed to match or improve the specifications of a commercial FDA approved stimulator (Natus Nicolet Cortical Stimulator, Natus Medical Inc., Pleasanton, CA; henceforth referred as the commercial stimulator).

Benchtop validation was performed to determine whether the BD-BCI stimulator accurately delivers stimulation parameters as commanded. Its accuracy was compared to that of a commercial stimulator to establish equivalence. To this end, the temporal responses to identical sets of commands will be compared and plotted to verify that the current/voltage level and pulse width scale correctly with the parameter sweeping across a 1 kΩ resistive load. The integrity of the signals was verified by time-aligning and overlaying the waveforms over a set period of time (e.g., 5 s) per sweeping current and pulse widths. The accuracy of current level, pulse frequency, pulse width, and train duration was assessed by comparing the user-requested command vs. measured values between the two stimulators.

### Design and validation of additional stimulator features

#### Charge density and impedance monitoring

The BD-BCI stimulator system was designed to conform to a stimulation charge density limit of 30 *μ*C/cm^2^, which was identified in previous animal studies as a safe limit (Agnew et al., 1986; McCreery et al., 1986, 1990; Kane et al., 2013). This safe limit was used in the first deep brain stimulator (DBS) approved in the US, the Medtronic Activa Tremor Control System (FDA, 1997) for essential tremor (FDA, 1997; Kuncel and Grill, 2004), as well as many other neural stimulator medical devices. The charge density (CD), expressed in *μ*C/cm^2^, is defined as: CD = I × PW / EA, where I: current (mA), PW: pulse width (ms), EA: exposed surface area of the electrode (cm^2^). CD was automatically calculated from the stimulation parameters commanded by the user *via* the GUI. As a protective measure, if the requested stimulation parameters exceed the 30 *μ*C/cm^2^ limit, the stimulator and GUI enter into a “lock” mode until the parameters are altered to safe levels.

To detect if electrode-brain contact for any stimulation channel is adequate, the load impedance between the two stimulating electrodes was derived as follows. A short test stimulation pulse was delivered across the electrode pair. The voltage across the electrode pair was measured by the microcontroller's onboard ADC. Similarly, a current sense resistor/amplifier (Texas Instrument, Dallas, TX) was used to measure the current. The impedance is then derived using Ohm's law. The stimulator was configured to “lockout” any channel with impedance >3 kΩ, indicating bad electrode-brain contact.

Impedance measurement was validated by comparing the true impedance (measured by a commercial digital multimeter, Kaiweets, HT206D) vs. the measured impedance (determined by the BD-BCI prototype) of the standard commercial through-hole resistors. Here, 8 different value of resistors between 330 and 1,500 Ω were measured five times each (this range is chosen as it represents the typical range of ECoG impedances (Sillay et al., 2013). A regression analysis between the true and measured impedance was used to assess the validity of the BD-BCI prototype.

#### Charge balancing

**Figure 3:**
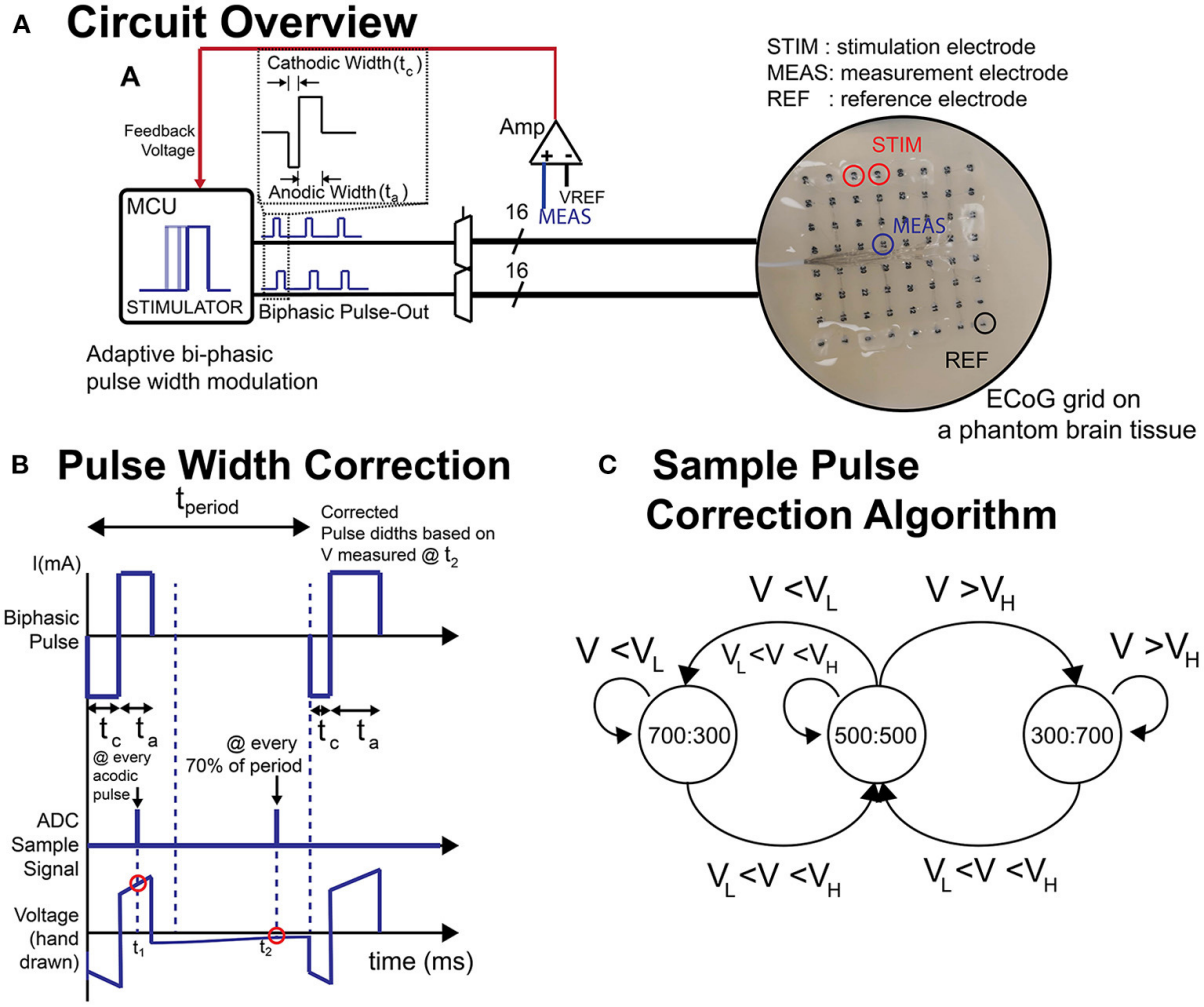
**(A)** Schematic of circuit and the ECoG grid in phantom brain tissue. **(B)** Illustration of biphasic pulse and the voltage sampling timing. **(C)** A sample state-machine implementing a threshold-based active charge balancing. t_*a*_, anodic pulse width; t_*c*_, cathodic pulse width; t_1_, sampling timing for impedance measurement; t_2_, sampling timing for active charge balancing (steady state voltage); V_*H*_, upper voltage threshold; V_*L*_, lower voltage threshold.

Passive and active charge balancing methods was used in the BD-BCI. The passive charge balancing was implemented by switching the H-bridge outputs at every off-duty cycle to GND to release residual charge accumulated at the stimulating electrodes. Active charge balancing utilized adjustments in stimulation pulse width to correct for any detected charge accumulation. Specifically, to measure the steady-state level potential at an electrode (given that residual voltage is directly related to charge), the voltage was sampled at 70% of every duty cycle between the pulses (Figure 3B). This was achieved using a buffer amplifier (Texas Instrument, Dallas, TX) and instrumentation amplifier (Texas Instrument, Dallas, TX) cascaded as in Figure 2B. Based on the measured voltage(V_*MEAS*_), the microcontroller applied a corrective pulse. A state-machine (Figure 3C) dictated how the ratio between cathodic and anodic pulse width are correctively adjusted (e.g., 700:300 μs, respectively) at the very next pulse cycle from the cycle of measurement (Figure 3B). According to this algorithm, when measured voltages exceed an arbitrary threshold, corrective pulses are generated to reverse the effect of the biased parameters to bring back the steady-state voltage below the thresholds.

To validate the charge balancing mechanism, 0 and –60 mV were set as the upper and lower tolerance thresholds (Figure 3). The system was tested to determine if the voltage (and thereby the charge) ever violates these thresholds. To this end, an anodic voltage offset was introduced to be corrected later with the corrective pulses (700:300 μs) according to the active charge balancing. The current and frequency were set to 12 mA and 200 Hz, respectively, and the stimulation was delivered continuously. Using a bioamplifer (MP150, Biopac System, Inc. Goleta, CA), we recorded the time-response of corrective pulses and the voltage at multiple neighboring electrodes in the ECoG grid when the active charge balancing function turned on and off over 20 s. The number of times that the voltage violated the upper or lower thresholds was determined.

The charge balancing and artifact mitigation tests were performed on a phantom brain tissue prepared as in Kandadai et al. (2012), Pu et al. (2020), and Sohn et al. (2020) so as to mimic the environment for implanted ECoG electrodes to test the stimulator and its responses. The phantom brain tissue was created by mixing 6 g of food-grade agar powder into 100 ml of warm water (85–90°C) with 50 mg of table salt. The solution was poured into a Petri dish and allowed to cool in a 4°C refrigerator overnight. During testing, the phantom was warmed to room temperature and a standard 8 x 8 ECoG grid (Ad-Tech, Oak Creek, WI) with platinum electrodes (4 mm diameter, 2.3 mm exposed diameter, 10 mm pitch) was placed onto the phantom. A thin layer of phosphate-buffered saline (PBS), which acts as a cerebrospinal fluid (CSF) analog, was poured on the Petri dish to ensure full contact and stimulation current was delivered across electrode pairs in a sweep across all parameters. This was first performed with the BD-BCI stimulator, and then repeated with the commercial stimulator. The stimulation responses across the stimulation channel were recorded by a commercial data acquisition system MP150 with a sampling frequency of 20 kHz. The accuracy and linearity of response will be assessed as the coefficient of determination (R^2^) between the commanded and measured parameters in a regression analysis.

#### Artifact mitigation

Artifact mitigation strategies (Zhou et al., 2018) have been proposed, including front-end techniques which focus on preventing saturation and rapid recovery in the amplifier and back-end techniques to recover the neural signals (Al-ani et al., 2011; Wichmann and Devergnas, 2011; Lu et al., 2012; Zeng et al., 2015). By reducing the stimulation artifact in the nearby channels, saturation of amplifier in the nearby brain area (e.g., on the motor cortex) can be prevented and the charge accumulation in the nearby electrodes can be further reduced. To reduce the impact of stimulation artifacts, an extra pole is added to the stimulating dipole which can be optionally activated to split the current into the extra pole to reduce the stimulation artifact amplitude. The extra pole can be selected with a multiplexer (A3 in Figure 2B) in a formation that two connected poles surrounds the other pole as an electromagnetic trap as in Figure 2B.

For assessment, the degree to which the artifact propagates to the nearby channels was verified by recording the time series data during stimulation with and without the artifact mitigation function. The percentage of artifact reduction was assessed by comparing the mean peak voltage of the artifacts before and after the method is applied.

### Design and validation of bi-directional BCI function in phantom brain

**Figure 4:**
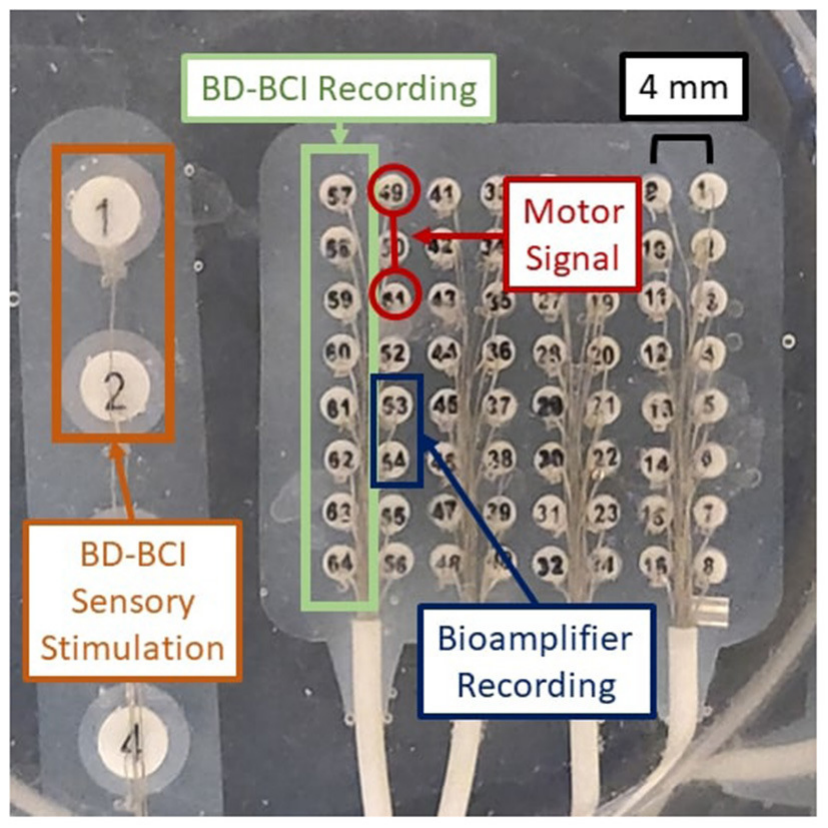
Experimental setup for BD-BCI operation testing on a brain phantom. Orange: BD-BCI output stimulation during periods of decoded move is applied to electrode 1 and 2 of the ECoG strip on the left. Red: External signal generator output acting as a mock motor signal is applied to electrodes 49 and 51 on the ECoG grid. Blue: Bioamplifier records channel 53 and 54 on the ECoG grid for acquisition purposes. Green: BD-BCI records ECoG channels 57 to 64 for decoding purposes.

A crucial aspect of BD-BCI operation is the ability to both perform decoding and stimulation. Here, the BCI software responsible for the decoding of ECoG signals was taken from our prior work in Wang et al. (2019). Since the BCI software component that performs online decoding of the brain signals to classify the ECoG data into either move or idle states in real time was already validated at the bedside in Wang et al. (2019), this study will focus on a benchtop test in a brain phantom to first ensure proper simultaneous operation of both the decoding and the interleaved stimulation. Using the same brain phantom as above, a 8 × 8 high density ECoG grid (2 mm electrode diameter, 4 mm pitch) was placed adjacent to a 1 x 6 single strip ECoG electrode (4 mm diameter, 10 mm pitch) positioned 10 mm (Figure 4) and immersed in PBS. Eight electrodes provided input ECoG signal to the bioamplifier in the BD-BCI system. A pair of electrodes on the strip was designated for sensory stimulation. Another electrode pair on the grid was designated to deliver simulated brain signal as a mock signal source for motor intention. This mock motor signal was generated by an external signal generator. During the training data collection procedure, the experimenter switched the signal generator on/off following 10-s-long move/idle cues over 6 trials (totaling 60 s) from the BD-BCI system base station (a sinusoidal wave, 100 Hz, 358 mVpp during move cues) while the BD-BCI acquires the simulated ECoG signals (at sampling frequency of 500 Hz). Once training data acquisition was complete, the BD-BCI system generated a decoding model as in Wang et al. (2019).

During online operation, the experimenter switched the signal generator on/off following 20-s alternating move/idle cues from the base station, with 10 move and 10 idle cues constituting one run. The BD-BCI system recorded ECoG signals as above and decoded 750 ms windows of simulated ECoG signal (250 ms stride). Since simultaneous electrical stimulation is likely to disrupt the ability to acquire ECoG signals properly during online BCI operation, the online BCI software was modified to interleave decoding and stimulation periods. We devised two stimulation schemes that coarsely simulate the sensory feedback of heel strikes in ambulation or the proprioceptive feedback during a leg swing. In the heel strike mode, while the move state is decoded, the BD-BCI delivers a 200-ms window of stimulation (ECoG signal acquisition and decoding paused during this time) every 1,200 ms. This mimics the process of delivering a tactile artificial percept with every step. Stimulation bursts were delivered as pulse-trains (50 Hz, 5 mA, 250 μs biphasic pulse width, 200 ms in burst duration) while the move state was decoded. The timing for decoded states and instructional cues were recorded and saved for performance analysis. The above procedure was performed again for the proprioceptive mode whereby short bursts of stimulation (50 Hz, 5 mA, 250 μs biphasic pulse width, 50 ms in burst duration every 250 ms) were delivered while the move state was decoded. For control, the above procedure was also performed without any stimulation. To evaluate the performance of each of these conditions, the percentage of correctly decoded states was calculated for each trial. To account for operator delays and decoded window offset, the cue and decoded state signals are lag optimized by calculating and applying a lag that maximizes the cross-correlation between the two signals. The decoding accuracy was calculated using these lag optimized signals.

### Bedside validation

After establishing the validity of basic functions of the BD-BCI stimulator, it is necessary to determine that it can elicit similar behavioral responses as the commercial stimulator. The commercial stimulator was clinically used for functional brain mapping as part of the epilepsy surgery evaluation (Ojemann et al., 1989; Roux et al., 2003) and acted as the benchmark target for our BD-BCI stimulator. Our BD-BCI stimulator acquired an abbreviated investigational device exemption (IDE) and IRB approval at Rancho Los Amigos National Rehabilitation Center (Downey, CA). Patients undergoing intractable epilepsy surgery evaluation with ECoG electrode implantation over the left M1/S1 area were recruited for this study. Functional brain mapping with the commercial system was performed after anti-epileptic drugs (AEDs) were restarted to identify eloquent brain areas so that they can be spared from resection. After the clinical functional brain mapping was completed, the procedure was repeated with the BD-BCI stimulator to assess the equivalency of the patient's sensorimotor responses. To this end, both the commercial and BD-BCI stimulator delivered bipolar stimulation with fixed frequency and pulsewidth, namely 50 Hz, 250 μs, respectively. The commercial system delivered 4 s pulse trains, whereas it was 2 s for the BD-BCI stimulator, as this shorter duration was sufficient to elicit sensorimotor percepts. To test for responses, stimulation was delivered starting at a minimum of 8 mA for the commercial stimulator and 3 mA for the BD-BCI stimulator. The current was increased incrementally by 2 mA for the commercial stimulator and by 1 mA for the BD-BCI stimulator, either until the patient reported a response, or sensorimotor response was observed, or afterdischarge activity on ECoG prevented further current increase, or the current was high enough to establish no clinical response existed at that particular channel. If the BD-BCI stimulator's maximum available current for the particular channel is lower than the current that meets any of the above conditions, the stimulation was stopped at that current. After each stimulation delivery, the patient was asked to report the perceived intensity, quality, and location of sensation or movement on the patient's body (the anatomical location was identified using a body map which divided the body surface into 45 compartments; Correia et al., 2015). The *in vivo* stimulation pulses delivered to the brain was measured and qualitatively compared between the commercial and BD-BCI stimulators using a commercial handheld oscilloscope (Siglent Technology, Shenzhen, China) connected in parallel to a stimulating electrode pair. The equivalency between the two stimulators will be quantified based on the percentage of matching anatomical localization of the responses between the two stimulators.

## Results

### Device fabrication

**Figure 5:**
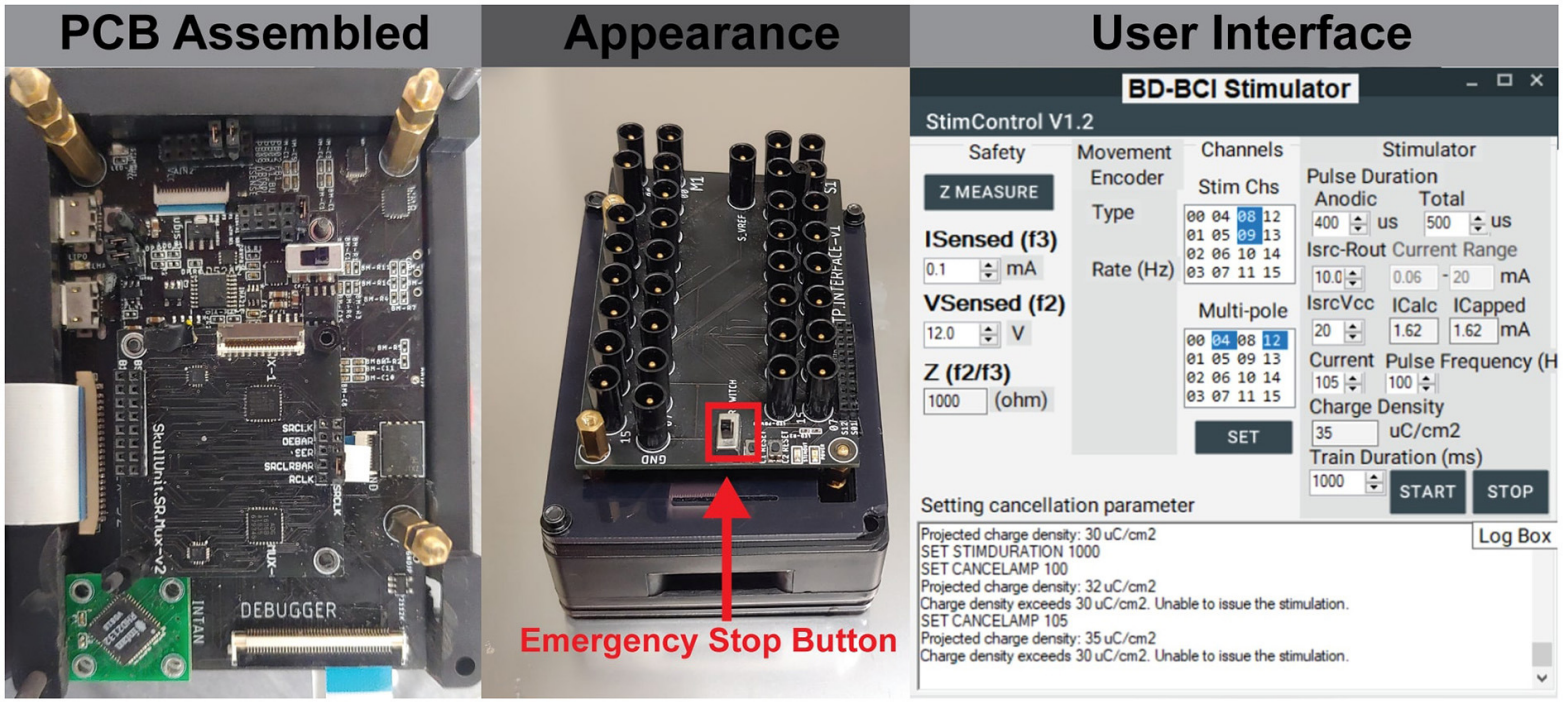
**(Left)** The assembled board which includes embedded system on modular printed circuit boards. **(Center)** The final appearance of the BD-BCI stimulator enclosed in a case, and 1.5 mm touch proof interfaces on the top for connecting ECoG electrodes. **(Right)** The GUI which provides user control of all stimulation parameters and electrode pairs selection.

The BD-BCI prototype PCBs were fabricated (Smart Prototyping, Shenzhen, China) and assembled. The system weight is 164 g, and the case's dimensions are 7×9×5 cm (similar in size to a Raspberry Pi; Figure 5). The GUI was implemented in C# as an add-on to our previous BCI GUI (Wang et al., 2019), which was executed on a desktop computer (Windows 10), and facilitated control of all stimulation parameters and features *via* the base station. The BD-BCI prototype system software pertaining to stimulation was implemented in C++ as an add-on to our previous BCI operating system (Wang et al., 2019), compiled, and deployed to the 3 MCU cores.

### Validation of basic stimulator function

**Figure 6:**
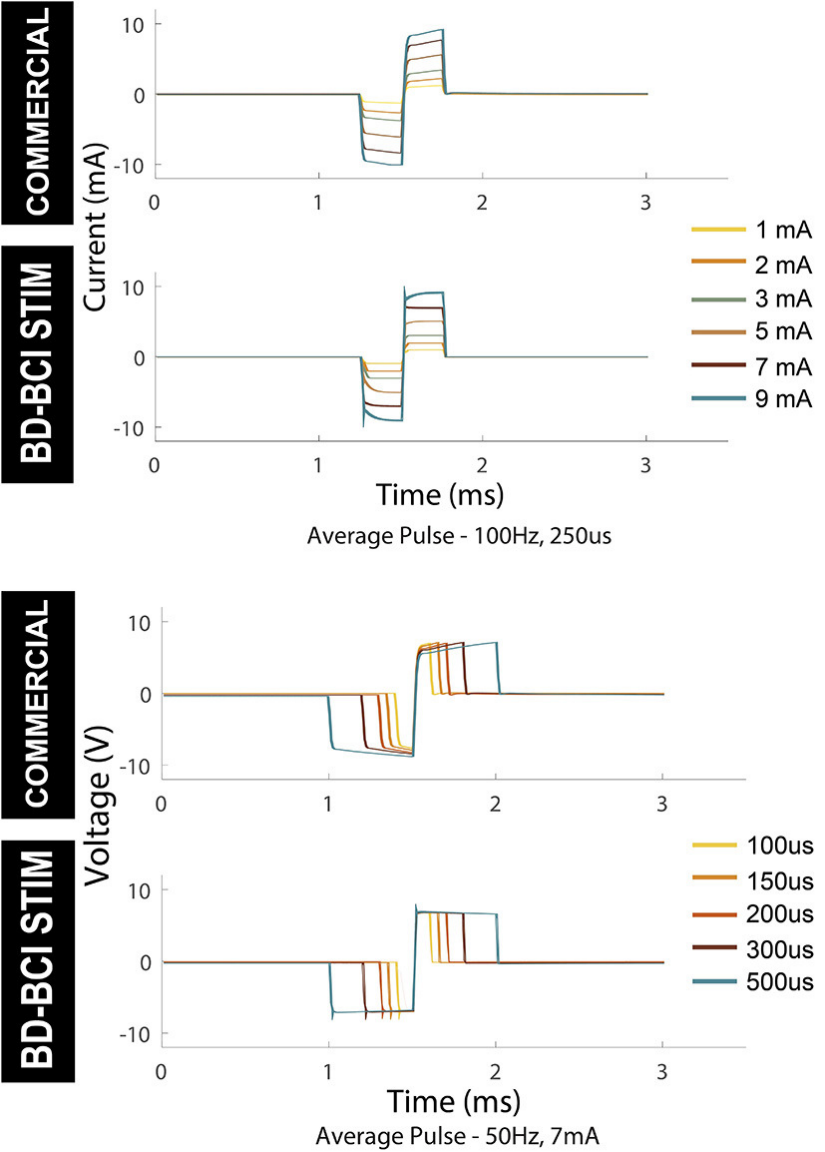
Output comparison of current amplitude and pulse width of the commercial and BD-BCI stimulator. Parameter sweeping of current and pulse-width are performed. The pulses in 5 seconds pulse-train are time-aligned and overlaid to demonstrate the accuracy and consistency of the pulse outputs.

**Figure 7:**
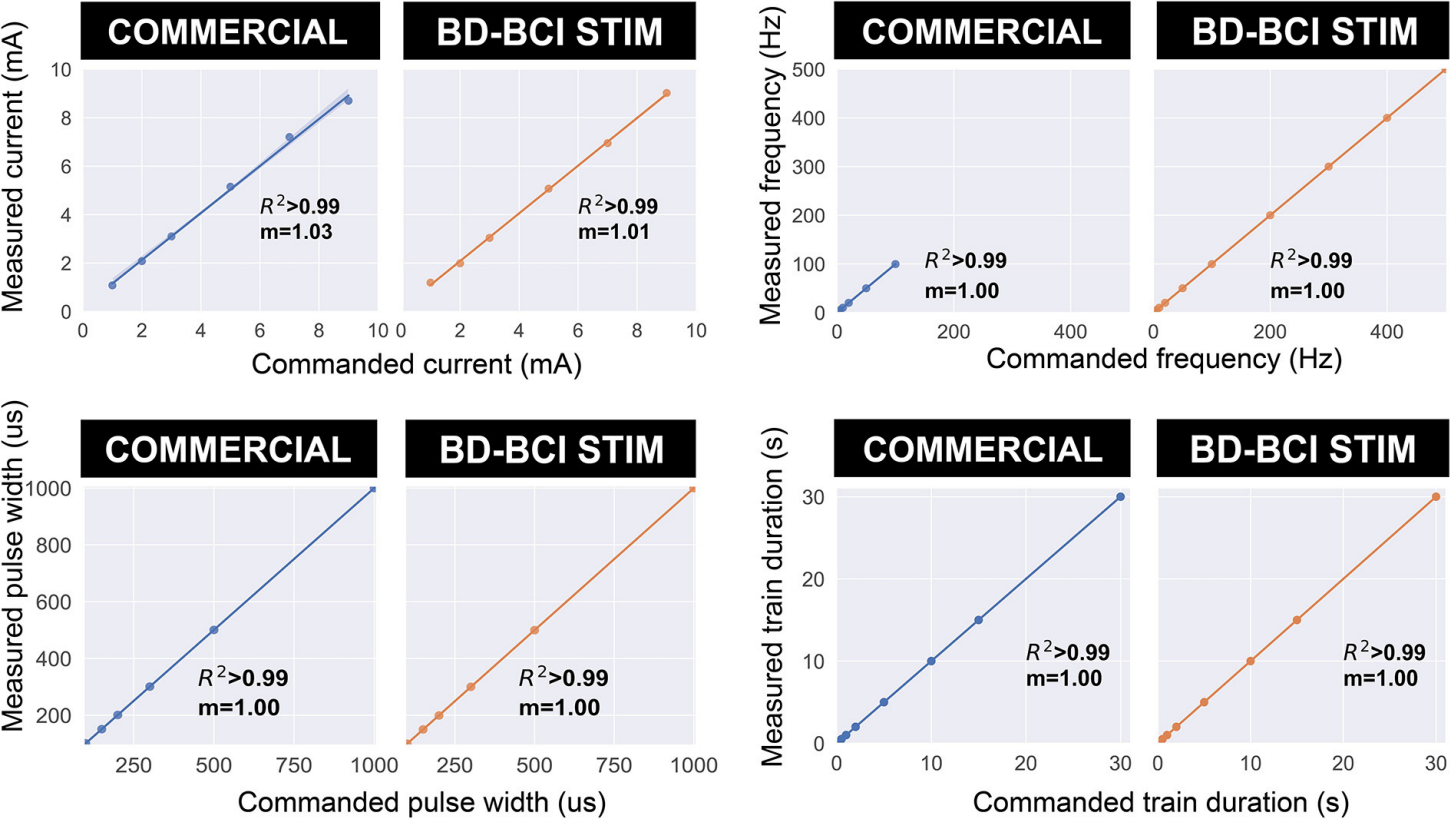
The accuracy of the commercial and BD-BCI stimulator. The comparison plots summarize the output equivalence of the BD-BCI stimulator to the FDA approved commercial stimulator. The measured controllability, outputs in the current amplitude, pulse width, pulse frequency, train duration are demonstrably equivalent. The labeled coefficient of determination (R^2^) indicates that the outputs are accurately controlled in all output categories. Specifically, the results indicate that both the commercial and BD-BCI stimulator deliver stimulation at exact parameters which were commanded.

Electrical stimulation was delivered to a pair of electrodes across a resistor as described in Section: “Design and validation of basic stimulator functions” by sweeping across current, pulse width, and frequency. Figure 6, top shows a comparison between the commercial and BD-BCI stimulator for 5 different levels of current commands while holding other stimulation parameters (100 Hz pulse frequency, 5 s train duration, 250 μs pulse width shown) across a 1 kΩ resistive load. The accuracy of the BD-BCI stimulator was compared with that of the commercial stimulator in Figure 7. There were high correlations between commanded and measured current for both the commercial (*R*^2^ > 0.99, intercept = –0.18, slope = 1.03) and BD-BCI stimulator (*R*^2^ > 0.99, intercept = –0.11, slope = 1.01), between commanded and measured pulse frequency for both the commercial (*R*^2^ > 0.99, intercept = –1.04, slope = 1.00) and BD-BCI stimulator (*R*^2^ > 0.99, intercept = 0.04, slope=1.00), between commanded and measured pulse width for both the commercial (*R*^2^ > 0.99, intercept = –1.14, slope = 1.00) and BD-BCI stimulator (R_2_ > 0.99, intercept = 0.28, slope = 1.00), and between commanded and measured train duration for both the commercial (*R*^2^ > 0.99, intercept = 0.00, slope = 1.00) and BD-BCI stimulator (*R*^2^ > 0.99, intercept = 0.00, slope = 1.00). These results demonstrate that the BD-BCI stimulator has highly accurate control of stimulation parameters and was output-equivalent to the commercial stimulator in controlling the current level, pulse frequency, pulse width, and train duration.

### Validation of additional features

#### Charge balancing

**Figure 8:**
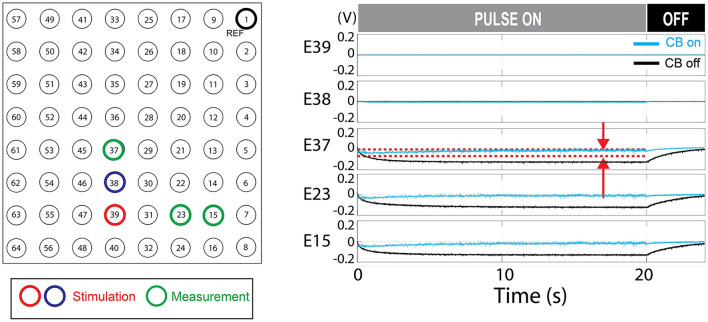
Charge balancing (CB) demonstration. The demonstrated example shows the effect of active charge balancing by comparing the time-aligned steady-state voltage traces for the charge-balancing function was ON and OFF (CB ON/CB OFF). The phantom tissue was stimulated with a pulse train that results in a slightly net negative bias. The voltage at the stimulating electrodes (E39 and E38) and the neighboring electrodes in the phantom tissue (E37, E23, and E15) are shown. The voltage was sampled at the steady-states, 70% of the duty cycles, as illustrated in Figure 3B. This time point is expected to represent the true steady state as in Figure 6. The stimulation was ON during the first 20 s and turned off. Due to the passive charge balancing, the steady-state voltage at E39, E38 are consistently zero. The other electrodes show the time-varying voltage traces which were suppressed when the charge balancing was ON. The red dotted lines mark the upper and lower threshold for the charge balancing which is only shown for E37. The stimulation frequency was 200 Hz and the current level was 12 mA. The CB feature is not present in the commercial stimulator.

Passive charge balancing ensured that there was no charge accumulation in the stimulation channels (E38, E39) although charge accumulation was seen in neighboring electrodes (Figure 8). The addition of active charge balancing mechanism maintained the steady-state voltage between the upper and lower thresholds 100% of times in neighboring electrodes in the test case [0 out of 400 k samples over the 20 s test period (Figure 8)].

#### Artifact mitigation

**Figure 9:**
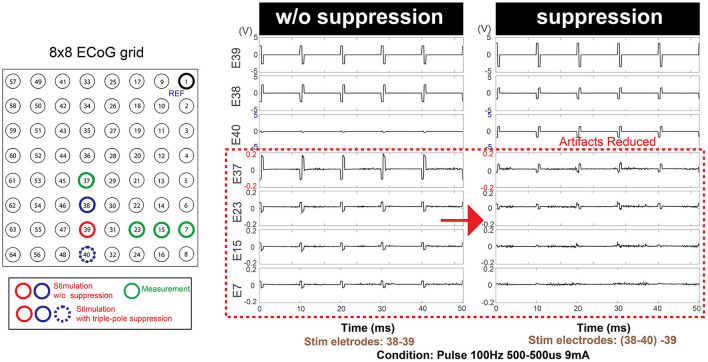
Additional optional safety feature: Artifact mitigation by triple-pole technique. An extra stimulation pole (E40) in addition to the existing stimulation dipole (E38-E39) contributed to the overall mitigation of the artifacts over the entire grid space. This feature is not present in the commercial stimulator.

The mean peak voltage of the stimulation artifact decreased by 64.5% in E37, 31.8% in E23, 43.1% in E15 and 44.1% in E7, providing case evidence that the technique can be effective not only to the nearest neighbors to the stimulation dipole but throughout the distant measurement sights. Figure 9 shows a representative example of artifact mitigation.

#### Impedance measurement and charge density monitoring

**Figure 10:**
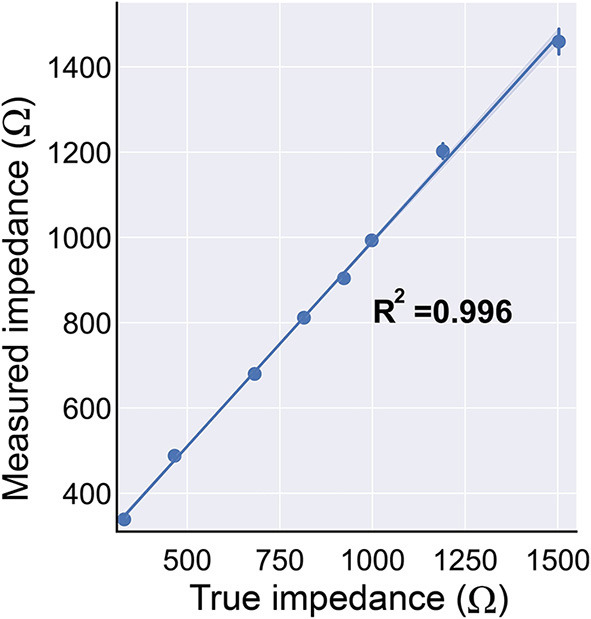
The BD-BCI on-board impedance measurement. The true impedance vs. the measured impedance demonstrates that the impedance measurement is accurate. The impedance monitoring ensures the electrode's contact with the brain is sufficient for safe stimulation.

The *R*^2^ between true and measured impedance was 0.996 (intercept = 28.96, slope = 0.962). An *R*^2^ and slope near 1.0 indicates that there were strong linear associations between the true and measured impedance (Figure 10). The prospective charge density was automatically calculated from the requested stimulation parameters, and the GUI successfully “locks” if it is > 30 *μ*C/cm^2^.

### Demonstration of the bi-directional BCI in phantom brain

**Figure 11:**
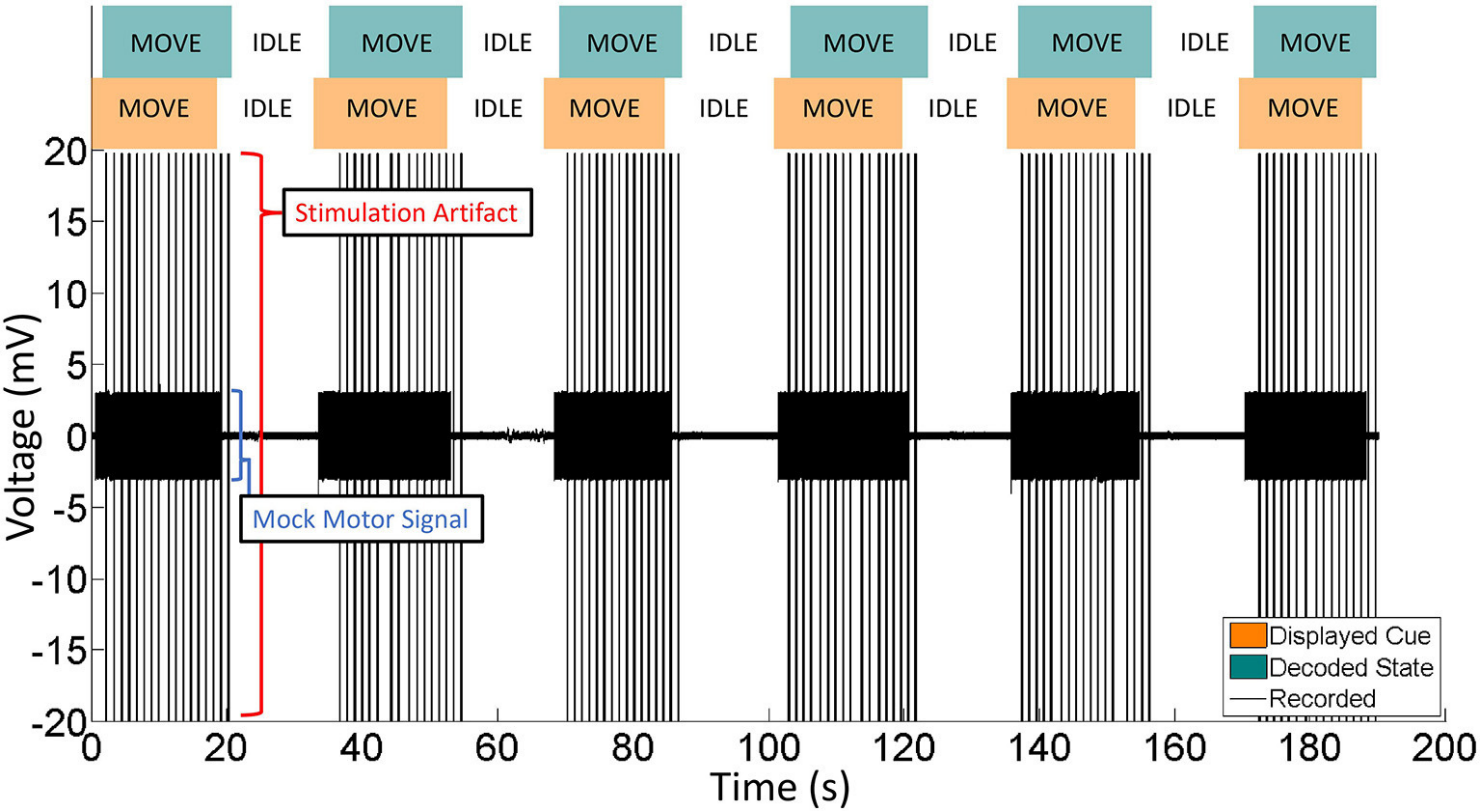
Representative portion of BD-BCI validation in phantom brain. Eleven move/idle segments are displayed. Orange: The cue, as output by the BD-BCI base station, acts as a ground truth. Green: The decoded state as determined by the BD-BCI. Black: The voltage data from one ECoG channel during BD-BCI operation. The spikes during move states are stimulation artifacts corresponding to stimulation bursts (5 mA current, 250 μs biphasic pulse width, 200 ms in burst duration) occurring at every simulated moments of heel-strike during imagined walking. The smaller voltage oscillations are the mock motor signal output by the function generator.

The decoding accuracy during BD-BCI was not affected by the addition of stimulation. The average and standard deviation percentages of correctly decoded states during the heel strike, proprioceptive, and no-stimulation modes during online BD-BCI operation are reported in Table 1. An example of the live decoding performance is shown in Figure 11. The latency between the cue and decoded state was 1,410 ± 250 ms. The variability in the latency was caused by uneven experimenter response time to the instructional cues, discrete interval in reporting of the decoded states due to non-overlapping decoding time windows (~400 ms), and jitter in the decoding and wireless transmission latency times. Based on the bioamplifier recording, artifacts from stimulation bursts occurred only during the decoded move states. This indicates that the BD-BCI system correctly applied stimulation and that the resulting artifacts did not disrupt the decoding results. During this BD-BCI operation, the system's power dissipation was measured to be ~377 mW.

**Table 1:** Accuracies for each of the BD-BCI stimulator stimulation modes in the phantom brain experiment.

**Stim. condition**	**Mean accuracy**	**SD**
No Stim.	0.968	0.023
Proprioceptive	0.968	0.022
Heel strike	0.968	0.023

### Bedside validation

**Figure 12:**
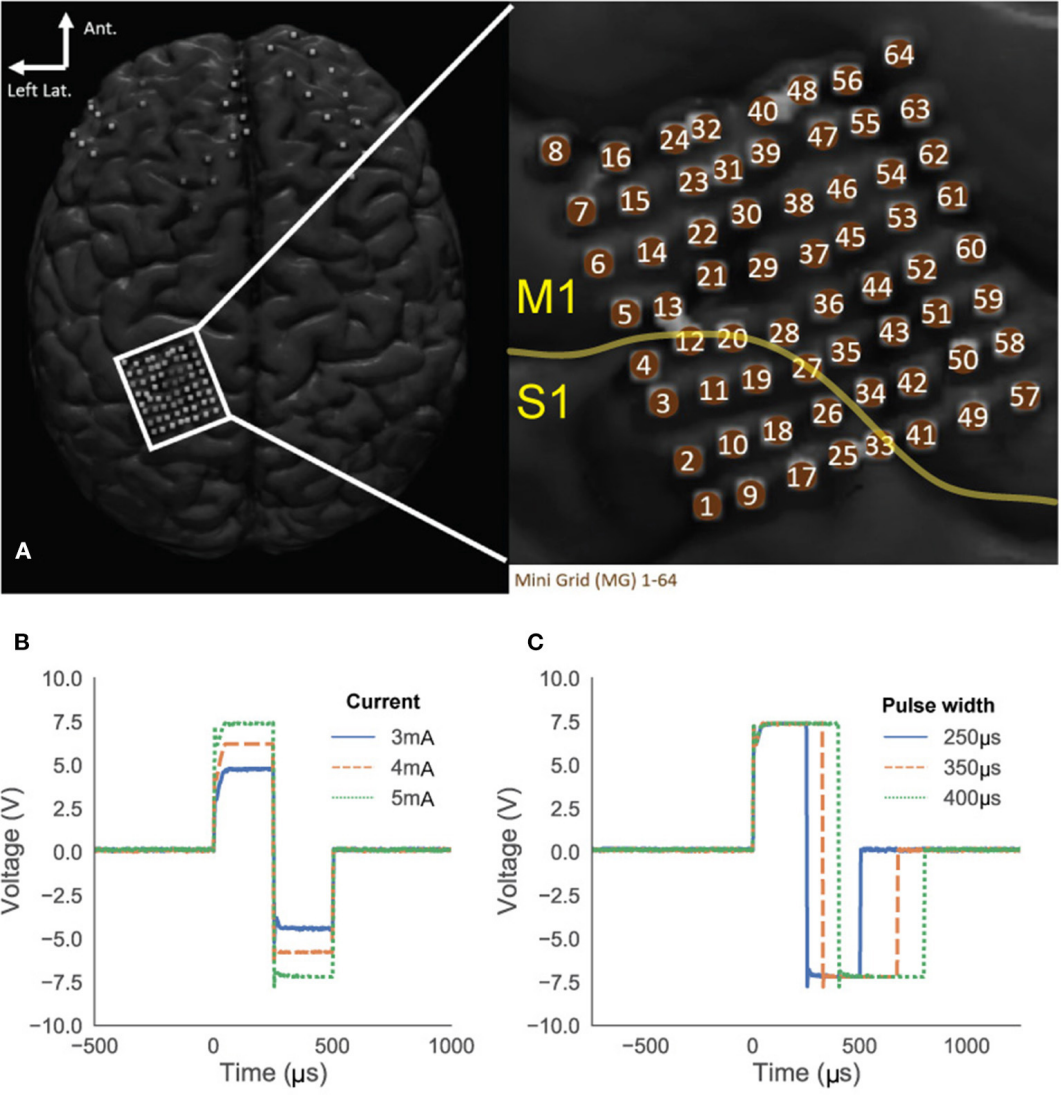
Subject's ECoG grid placements **(A)**. The yellow line indicates the estimated central sulcus which divide primary motor cortex (M1) and sensory cortex (S1) suggesting that the most of the grid space may elicit motor response. **(B, C)** Stimulation pulses in the patient's brain recorded from electrode 49 and 50. Amplitude sweeping from 3 to 5 mA **(B)** and pulse width sweeping from 250 to 400 μs **(C)**.

**Figure 13:**
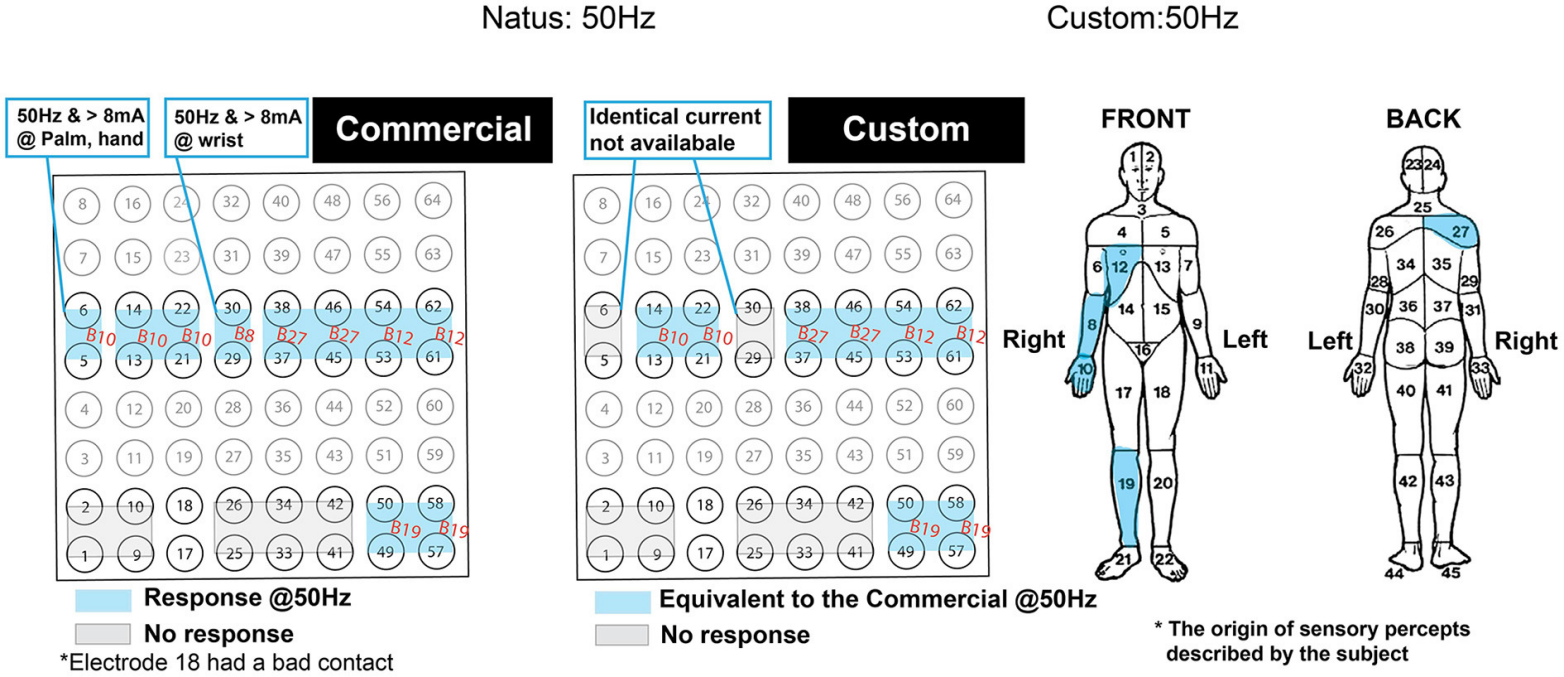
Side-by-side comparison of the patient's self-reported response from the functional brain mapping performed at 50Hz stimulation frequency. Blue highlighted regions indicate motor responses and are annotated with body region within which the response was originated. The gray zone indicates the region with no response. The response was due to 2 s of stimulation with the commercial **(left)** and the custom system **(right)**. The compartmentalized body map on the right was used as a reference for the annotated body regions. The orientation of the grid is displayed to match the Figure 12A.

A single epilepsy patient (female, age 42) undergoing epilepsy surgery evaluation provided informed consent to participate in this study. The ECoG grid locations were identified by MRI-CT image fusion as described in Wang et al. (2013) (electrode placement dictated by clinical needs). Figure 12A shows the axial view of the electrodes connected to the BD-BCI stimulator. The mapping procedures with the BD-BCI stimulator were performed the day after clinical mapping was conducted with the commercial stimulator. Given the patient's limited availability, only half of the grid space was mapped with the BD-BCI stimulator, including electrodes in rows starting with electrodes 1, 2, 5, and 6. Bipolar stimulation with electrode pairs in vertical axes (e.g., 1–2, 9–10, 17–18, ...) was performed. Figures 12B, C shows sample stimulation pulses measured *in vivo* at the channel formed by electrodes 49–50 across a sweep of current and pulse width. Figure 13 summarizes the side-by-side comparison of the patient's response to the functional brain mapping by the commercial and BD-BCI stimulator, and demonstrates identical response between the two stimulators. All responses were motor, and neither system was able to elicit any patient response in electrodes overlying S1. In two channels, 5–6 and 29–30, the current parameters used to elicit a response with the commercial stimulator (>8 mA) were not available in the BD-BCI stimulator due to higher impedance (>1.7 kΩ) since the BD-BCI stimulator has lower output voltage (13 V) compared to the commercial stimulator (24 V), all of the matching grid space elicited response to the equivalent body location. Thirteen out of 13 electrode pairs (100%) produced responses to the same body location. Throughout this study, the patient received no additional AED, was seizure-free with no after-discharges or epileptiform discharges observed on ECoG signals, and expressed no subjective complaints.

## Discussion

The results above demonstrate that our custom stimulator has fully programmable access to all stimulation parameters, is equivalent to an FDA-approved commercial stimulator, is likely safe for long term use, and can operate simultaneously to BCI decoding in an interleaved fashion. Specifically, the cortical stimulator module behaved identically to an FDA-approved commercial stimulator and is fully programmable with highly accurate parameter control. Most importantly, the BD-BCI stimulator could elicit identical sensorimotor responses to those of the commercial system in bedside testing. Online BD-BCI operation was also validated in a phantom brain. Finally, safety features in the BD-BCI stimulator make it suitable for potential future use in humans (Table 2B). Overall, successful integration of a fully programmable stimulator and decoder into a single embedded system represents an important BD-BCI development milestone. This provides a testbed platform that can enable validation of a variety of prospective BD-BCI applications within realistically approximated constraints of a future fully implantable system. The low cost nature of our system (~$1,000 in components and fabrication cost, and ease of software development given access to open-source development kits for off-the-shelf MCUs) helps to reduce the barrier of entry into the field of invasive BCIs, particularly in the area of BD-BCIs where commercial stimulator equipment may cost orders of mangitude higher (> $20–100 K) and yet possess less flexibility. These aspects will be discussed in further detail below.

**Table 2:** The output equivalency table between the FDA approved commercial stimulator **(A)** and the BD-BCI stimulator **(B)**.

	**A**	**B**
**Output features**	**Commercial**	**BD-BCI STIM**
Bi-phasic current pulse	✓	✓
User-configurable current level	✓	✓
User-configurable pulse frequency	✓	✓
User-configurable pulse width	✓	✓
User-configurable train duration	✓	✓
User-selectable electrode pairs	✓	✓
Activation stimulation indicator light	✓	✓
Current output (mA)	0–15	0–15
Pulse frequency (Hz)	1–100	1–500
Pulse width (μs)	100–1,000	1–1,000
Train duration (s)	0.1–30	0.1–60
Max stimulation charge (μC)	15	15
**Additional safety features**		
Impedance monitoring	N/A*	✓
Artifact mitigation	N/A	✓
Charge-monitoring/balancing	N/A	✓
Charge density monitoring (μC/ph/cm^2^)	N/A	✓

The checkmark denotes the presence of the specific output feature. The table indicates that all the output features from the FDA approved commercial system are present in BD-BCI stimulator. Additionally, enhanced safety features: impedance monitoring, artifact mitigation, charge monitoring/balancing, charge density monitoring are present in BD-BCI stimulator, but absent in the commercial system. N/A, Not Available. *Feature may exist, but not accessible to users.

Both benchtop and bedside tests comparing the FDA-approved commercial stimulator and the BD-BCI stimulator revealed nearly identical concordance between the two systems across all features (Table 1). The equivalence with an FDA-approved commercial cortical stimulator indicates that our fully programmable and miniaturized stimulator architecture can readily gain regulatory approval to safely undertake future human studies in BD-BCI applications or other closed-loop neural stimulation studies. This is further bolstered by integration of the stimulator with our pre-existing BCI system from Wang et al. (2019) to achieve BD-BCI functionality using an interleaved decoding and stimulation approach. This BD-BCI approach was also validated in benchtop phantom brain, whereby decoding accuracy was not affected by stimulation. While bedside BD-BCI testing with human subjects was not performed here, we expect that the inclusion of interleaved stimulation should not fundamentally affect the validity of the decoding mechanism (previously validated at the bedside in Wang et al., 2019).

Nevertheless, having validated the critical functions and safety of the system, future studies can now be readily performed in humans to determine how the addition of sensory feedback affects the user's ability to operate the BCI. While only 2 modes of feedback stimulation are tested here, the system can be readily configured to deliver other customized modes of stimulation for BD-BCI operation. However, interleaving between periods of sensory stimulation and BCI decoding introduces time gaps where no decoding occurs due to stimulation, and it will be necessary to determine if these gaps disrupt users' ability to achieve good online BCI control. Note that while only square wave stimulation was tested here, the system also has the flexibility to deliver custom waveforms by streaming amplitude commands to the digital rheostat (see Figure 2) throughout the stimulation duration. In addition, the BD-BCI's wireless communication capability enables it to respond to external sensors (e.g., tactile, pressure, vibratory, or even gyroscopic) so as to elicit a wide variety of sensory percepts. Although our system only has 1 stimulator unit, it can potentially be configured to deliver multiple percepts at multiple sensory locations. Given the very short duration of a typical stimulation pulse (on the order of μs), delivering multiple sensory percepts could be achieved by rapidly switching between different channels and stimulation parameters through a combination of switching commands to the multiplexor and digital rheostat. Finally, the ability to both decode and stimulate to deliver sensory percepts in a near real-time fashion with an “all-inclusive” embedded system is a crucial developmental milestone. By eliminating reliance on off-the-shelf systems with limited accessibility and portability, as seen in previously reported BD-BCI systems (Raspopovic et al., 2014; Weiss et al., 2018; Flesher et al., 2021), this system provides a benchtop system to safely test BD-BCI concepts in humans within realistic, approximate constraints of a future implantable system. More specifically, BD-BCIs using off-the-shelf systems often take advantage of the high computational bandwidth afforded by full sized computers to perform all the necessary decoding and stimulating functions. Such computationally expensive methodologies may subsequently not translate well into a scaled down fully implantable system. Without first adapting applications with computationally intense decoders to execute successfully on embedded testbeds as the current system, such approaches may find themselves without an optimal clinical translational path forward. Alternatively, they would be forced to tether their system to external computers for decoding, which would result in mobility limitations.

The successful inclusion of charge monitoring, charge density limits, and charge balancing/dissipation features helps to facilitate potential long-term BD-BCI operation in the future. These features collectively minimize charge accumulation at the stimulation channel, which in turn prevents damage to brain tissue and neighboring electrodes. Specifically, our BD-BCI system included a combination of passive and active charge balancing measures that were highly effective in removing voltage offsets (and thereby charge accumulation) that could develop in the stimulation electrodes and neighboring electrodes (Figure 8). This simple threshold-based active charge balancing was sufficient, and requires minimal computational burden so as to reserve processing power for more computationally demanding processes, e.g., decoding. The CD limit of 30 *μ*C/cm^2^ adopted in this study is in accordance to the a level empirically determined as safe in the previous animal studies and commonly used in FDA approved neural stimulation devices (Agnew et al., 1986; McCreery et al., 1986, 1990; Kane et al., 2013). CD monitoring and the highly accurate impedance monitoring together ensures the safe delivery of the intended charge. Such CD limits and accurate CD monitoring help to mitigate electrochemical changes at the electrode-tissue interface, which may cause damage to the electrodes and the brain tissue, particularly if an electrode is polarized during a stimulus pulse to an extent of causing irreversible redox reactions. This could also in turn electrolyze water, leading to pH changes, gas formation, and electrode degradation (Merrill et al., 2005; Cogan, 2008). By comparison, advanced commercial deep brain stimulators are equipped only with passive charge-balancing mechanisms (Foutz and McIntyre, 2010; Akbar et al., 2016; Almeida et al., 2017), and BD-BCI systems reported in the literature do not include any such advanced safety features. Given limited access to subjects undergoing ECoG electrode implantation and that basic safety testing of the stimulation module was our highest priority, these long term safety features were not validated at the bedside in this study. However, since basic safety of the BD-BCI stimulation module and its equivalence to an FDA-approved stimulator in an short term recording scenario have been established as above, validation of these additional safety features in future longer-term studies can now readily be undertaken. Finally, the approach taken here can provide guidance to other researchers to safely establish equivalence of their own custom brain stimulator systems to FDA-approved stimulators.

The tri-polar artifact mitigation technique reduced the mean peak voltage of the stimulation artifact by a range of 31.8–64.5%. This front-end approach reduces the risk of amplifier saturation, which can be an important factor in facilitating future “full duplex” BD-BCI function. Specifically, since decoding of M1 signals need to occur simultaneously or near-simultaneously to S1 sensory stimulation, artifacts due to electrical stimulation would likely confound or disrupt proper decoding. Although complete elimination of artifacts was not achieved here, mitigation of the artifact magnitude will reduce amplifier settling times and facilitate easier artifact removal for any future back-end methods (Wichmann and Devergnas, 2011; Lu et al., 2012; Limnuson et al., 2013; Zeng et al., 2015; Zhou et al., 2017). Whereas, the current BD-BCI system alternates between periods of decoding and stimulation, this tri-polar or similar multi-polar stimulation approach combined with software-based removal of residual artifact could enable a “full-duplex”-like simultaneous decoding and stimulation. Finally, whether the tripolar stimulation elicits similar behavior responses as does the conventional bipolar counterpart was not determined at the bedside since this study needed to first establish the basic safety of the stimulator module.

Further optimization with more advanced PCB design and smaller discrete components could drastically reduce the current BD-BCI benchtop prototype. In addition, translating the design into a custom IC or system-on-chip (SOC) may further reduce the footprint to a size that is suitable for a fully implantable system. Such optimiziation can also help reduce the system power dissipation below the 377 mW seen here, which is particularly important for future implantation. Despite this level of power disspitation likely falling within acceptable and safe range of heat dissipation within the human body (Park et al., 2016), it may necessitate battery charging approximately once per day, which can be viewed as a hassle by potential users. While some custom IC stimulators aimed toward BD-BCI application can be seen in Pu et al. (2021a,b), these preliminary works have not reached a point of being ready for equivalency testing with FDA-approved commercial stimulators at the benchtop and bedside. Further work in IC or SOC development to reduce this embedded BD-BCI to an fully implantable form factor is likely justified only once human studies using a benchtop “board-level” prototype as the current system demonstrates robust function in humans. Such a developmental pathway is comparable to what is taken by medical device companies. Currently, some commercial neural recording and stimulating devices, e.g., deep brain stimulators, have been repurposed for BCI applications (Vansteensel et al., 2016). This is made possible *via* software modifications and external attachments that enable “off-board” signal processing and decoding on an external computer. However, repurposing such systems for fully invasive BD-BCI operation has not yet been reported. Due to their primary purpose in the treatment of unrelated neurological diseases, such systems have severe limitations in the number of recording channels. By comparison, our BD-BCI system has the computing capacity to readily use up to 32 channels as in Wang et al. (2019) channels. More importantly, the BD-BCI system has the capability to operate independently without reliance on external systems for signal processing once the system has been configured. Additional future work may also examine how such a system could be applied in non-BCI applications, such as restoring somatosensory percepts for amputees, actuating prosthetic limbs, or for other forms of paralysis or sensory loss.

### Limitation

Although the prototype BD-BCI system matched the FDA-approved commercial stimulator in the featured specifications and demonstrated superior safety features that are important for the future development of the fully-implantable system, only one patient was available for bedside testing (limited accesibility to this patient population throughout the COVID-19 pandemic, and not all patients undergoing ECoG will get comprehensive stimulation mapping for such a comparison to be readily performed). Although only motor responses could be elicited, the commercial stimulator was likewise also not able to elicit any sensory responses. This limitation is likely due to the ECoG grid placement being primarily on the motor cortex. However, the fact that both systems elicited similar behavior responses implies our custom BD-BCI stimulator will likely be able to elicit sensory percepts whenever ECoG electrodes are placed over the primary sensory cortex. Furthermore, having achieved stable and reliable responses in benchtop and bedside tests described above, we do not expect the electrical behavior of the system to deviate from the above findings if applied to other human subjects. Implementation of truly simultaneous decoding and stimulation, i.e., “full-duplex” operation, was not undertaken here and is out of the scope of this study. This represents the most challenging development aspect of a BD-BCI system since robust real-time hardware and software artifact removal methods will likely be required to preserve the original ECoG signal in the presence of stimulation artifact so as to achieve accurate decoding. Even commercial closed-loop neural technologies, such as deep brain stimulators are affected by this problem. Due to the broad spectral band of stimulation artifact, purported closed-loop operation, i.e., Medtronic Percept DBS, will be limited to input from the low frequency bands, e.g., β band. As such, the interleaved / decoupled stimulation and decoding approach taken here is similar to that taken by other BD-BCI studies (O'Doherty et al., 2011; Weiss et al., 2018; Young et al., 2018).

## Conclusion

The fully programmable BD-BCI stimulator designed here is output-equivalent to the FDA-approved commercial stimulator and possesses additional safety features and functions that can facilitate chronic use. To date, there are no miniaturized and fully programmable cortical stimulator with such a safety profile. Achieving equivalency to an FDA-approved commercial stimulator enables future work to focus on further miniaturization and clinical applications in true BD-BCI operation, i.e., real-time brain-control of prosthetic limbs with simultaneous artificial sensory feedback.

## Data availability statement

The datasets presented in this article are not readily available because the human data from the participant of this study may not be disclosed to the public. However, the benchtop analysis data may be available upon reasonable request. Requests to access the datasets should be directed to and@uci.edu.

## Ethics statement

The studies involving human participants were reviewed and approved by Ethics Committee is UCI and Rancho IRB. The patients/participants provided their written informed consent to participate in this study.

## Author contributions

WS, PW, CL, RA, HP, ZN, and AD conceived and designed the study. WS, PW, JL, and AD developed the software and hardware of the BD-BCI system. WS, PW, and JL performed the benchtop tests and data analysis. WS, JL, PW, SS, MA, HG, and AD conducted the bedside tests. WS drafted the manuscript. AD critically reviewed the manuscript. All authors reviewed and approved the final manuscript.
